# Effectiveness of disinfectant solutions associated or not with brushing on the biofilm control of a 3D printed-denture base resin^*^

**DOI:** 10.1590/1678-7757-2023-0104

**Published:** 2023-07-17

**Authors:** Thaís Soares Bezerra Santos NUNES, Marcela Dantas Dias da SILVA, Sabrina Romão Gonçalves COELHO, Hamile Emanuella do Carmo VIOTTO, Ana Carolina PERO

**Affiliations:** 1 UNESP Faculdade de Odontologia Departamento de Materiais Dentários e Prótese Araraquara São Paulo Brasil Universidade Estadual Paulista (UNESP), Faculdade de Odontologia, Departamento de Materiais Dentários e Prótese, Araraquara, São Paulo, Brasil.

**Keywords:** Printing, Three-Dimensional, Denture bases, Biofilms, Denture cleansers

## Abstract

The formation of biofilm on denture bases is a recurrent clinical problem that favors the development of denture stomatitis. The effectiveness of a hygiene protocol in a 3D-printed denture base resin is still uncertain.

**Objective:**

To evaluate of the effectiveness of immersion, associated or not with brushing in a soap solution, on the biofilm control of a 3D-printed denture base resin.

**Methodology:**

Specimens of denture base resins [Cosmos Denture (COS) and Classico (CLA/control)] were contaminated in vitro with Candida albicans and immersed in sodium hypochlorite 0.25% (SH, alkaline peroxide) AP, chlorhexidine digluconate 2% (CD or PBS-Control), associated or not with brushing with 0.78% Lifebuoy soap. Roughness was evaluated before and after brushing and immersion. The effectiveness of the protocols was assessed by CFU/mL, cellular metabolism (XTT), scanning electron microscopy (SEM), and confocal scanning laser microscopy. Data were analyzed by T student, ANOVA/Welch, and Tukey/Gomes–Howell pos-hoc tests (α = 0.05).

**Results:**

CLA showed greater roughness than COS. CFU/mL and XTT were higher in COS resin with a higher hyphae formation. Immersion in SH and CD eliminated CFU/mL and reduced XTT for both resins, associated or not with brushing. AP reduced CFU/mL only when associated with brushing.

**Conclusions:**

The biofilm on the 3D-printed resin was thicker and presumably more pathogenic, regardless of its smoother surface. Immersions in SH 0.25% and CD 2% are effective hygiene protocols for both resins, associated or not with brushing. AP should be recommended when associated with brushing with a Lifebuoy 0.78% solution.

## Introduction

Digital technology for the manufacture of denture bases using the additive technique (3D printing) has offered advantages regarding financial savings, greater number of pieces per impression, printing of larger pieces, complex geometries, faster production, and less waste.^1,2^ However, the studies that evaluated the accuracy of the additive technique^3-5^ lack evidence on the mechanical and biological behavior of 3D-printed resins.

*C. albicans* is the main opportunistic pathogen associated with denture stomatitis.^6^ Moreover, the ability *C. albicans* to adhere and form structured biofilms on the resin of the denture base have been considered one of the main factors responsible for the development of the disease.^7^ That can evolve into a systemic infection and result in candidemia, a nosocomial infection with a high mortality rate.^8^

An effective hygiene method is essential for the removal of biofilm from dentures. Among such methods, (1) mechanical, by brushing, (2) chemical, by immersion in substances, and (3) physical, by microwave irradiation, laser, and photodynamic therapy stand out.^9^ The combination of mechanical and chemical methods has been recommended especially for denture wearers and older adults with compromised manual dexterity and visual accuracy.^9^

Sodium hypochlorite (SH) and alkaline peroxide (AP) are the most used substances for immersions from complete denture.^9,10^ Immersion in 0.25% SH has been indicated as an effective protocol for the control of *Candida spp*^11-13^ and AP has been recommended due to its low cytotoxicity^14^ and absence of residual odor.^15^ A 10-minute immersion in 2% chlorhexidine digluconate (CD) provides long-lasting disinfection in complete dentures colonized by *Staphylococcus aureus.*^16^ Among the liquid soap solutions, Lifebuoy stands out as an accessible alternative, since it is effective for the control of *C. albicans* and *C. tropicalis* biofilms on acrylic resin samples,^17^ free of cytotoxicity, and without changes in physical and mechanical properties.^18,19^

The 3D technology in dentistry is an essential innovation. Due to the lack of evidence on an adequate hygiene protocol for 3D-printed denture base resins, this study aimed to propose an effective hygiene protocol for the control of the biofilm formed on the denture bases fabricated by the 3D-printing method. Considering that the main users of removable dentures are older individuals with low manual dexterity, the results of this study are even more relevant. The null hypothesis is that the protocol will not affect the biofilm formation and surface roughness, regardless of the denture base resin.

## Methodology

**Figure 1:**
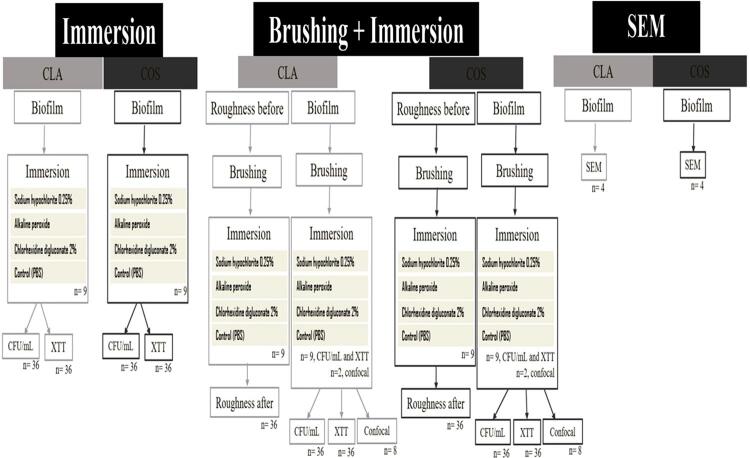
Flowchart for distribution of specimens

Figure 1 shows the manufacture and random distribution of circular specimens of a 3D-printed denture base resin (Cosmos TEMP, Yller Biomateriais SA, Pelotas, RS, Brazil, n=192) and a conventional heat-polymerized one (Classico, Artigos Odontológicos Clássico Ltda, São Paulo, SP, Brazil, n=192).

### Preparation of specimens of the heat-polymerized denture base resin - CLA

Metal matrices with 10 mm diameter and 1.2 mm thick cavities were included with a dental stone sandwiched between two glass plates blasted with aluminum oxide for the standardization of the roughness of the samples in ± 3.0 μm,^20^ simulating the internal denture base surface. The denture base resin (CLA) was manipulated according to the manufacturer’s recommendations. The hydraulic pressing was performed in two stages^21^ and an automatic tank (Solab SL-150, Solab Equipment for laboratories Ltda, Piracicaba, SP, Brazil) was used for the polymerization in water bath (90 min at 73°C + 30 min at 100°C).

### Preparation of specimens of the 3D-printed denture base resin - COS

The virtual design of the 10 mm diameter and 1.2 mm thick specimens was performed in Adobe Meshmixer software. The specimens were printed on a Flashforge Hunter DLP Resin 3D Printer (Zhejiang Flashforge3D technology Co., Jinhua City, ZheJiang, China) (LED, λ=405nm and 50 µm layer thickness) at a 0° angle to the platform,^22^ washed in 99% isopropyl alcohol (5 min), and kept in UV light in a Curing Box (Done 3D, Ribeirão Preto, SP, Brazil) for 10 min.

The roughness (Ra) (μm) of the CLA and COS specimens was evaluated before the tests by a digital profilometer (SJ-400-Mitutoyo Corporation, Tokyo, Japan) of 0.01 μm precision. The specimens were stored in distilled water at 37°C for 50±2 h before the tests.

### Strains and Culture Conditions

Standard strains of *C. albicans* (SC5314) were reactivated in Sabouraud dextrose agar with 0.05 mg/mL of chloramphenicol (Acumedia Manufacturers Inc., Baltimore, Maryland, USA) at 37°C for 48 h. Five colonies were transferred to TYE (Tryptone and Yeast Extract) and incubated overnight at 37°C. The fungal suspension was diluted (1:10) in fresh TYE and incubated at 37°C until the optical density at 540 nm had reached the mid-log phase of growth (0.649±0.0181), obtaining a 1.45×10^7^±3.84×10^6^CFU/mL concentration.

### Mature biofilm formation

The specimens were disinfected by exposure to UV light,^23^ transferred to 24-well plates containing half fungal suspension and half TYE + 1% glucose and incubated for 90 min at 37°C (adhesion phase)^24^. Next, the specimens were washed with PBS, transferred to a new 24-well plate with TYE medium + 1% fresh sucrose, incubated for 48 h at 37°C to obtain mature biofilms, and washed once with PBS. The mature biofilm formation was repeated on three occasions in triplicate for CFU/mL (n=9)^25^ and XTT (n=9), on one occasion in duplicate for confocal microscopy (n=2), and in triplicate for SEM (n=3).

### Hygiene protocols

The specimens were immersed for 10 min in 24-well plates for the immersion protocols with the respective hygiene solutions: 0.25% SH, AP, 2% CD, and PBS-Control group. Moreover, the specimens were subjected to brushing with a 0.78% Lifebuoy soap solution^18^ and immersed as previously reported for the brushing + immersion association.

Brushing was performed in ten cycles on a mechanical brushing machine in a laminar flow cabinet.^15^ All brushes (Sorriso - Colgate Palmolive Ind.e Com. Ltda., São Paulo, SP, Brazil) and devices where the specimens were inserted were previously sterilized in UV light for 20 min.^15^

### Count of colony forming units per milliliter

After the hygiene protocols, mature biofilms adhered to the specimens were scraped with a pipette tip in 24-well plates with PBS and tenfold serial dilutions in PBS were plated in duplicate on SDA and incubated at 37°C for 48 h for CFU/mL.

### Evaluation of cell metabolism (XTT)

After applying the protocols, the specimens were transferred to a 24-well plate containing an XTT solution of 2.5 mg/mL (0.5 μL of menadione and 5 mL of XTT)^26^ and incubated at 37°C in the dark for 3 h. An aliquot of each well was transferred in duplicate to a 96-well plate Elisa reader at 492 nm.^26^ The duplicate mean corresponded to the XTT value of each specimen.

### Analysis by scanning electron microscopy (SEM)

Specimens contaminated with mature biofilms were fixed in 2.5% glutaraldehyde (1 h/room temperature),^27^ washed twice with PBS, and dehydrated in 70% (1 h), 90% (1 h) and absolute (five times of 30 min each) ethanol solutions.^27^ Next, they were stored in a desiccator with silica for seven days,^27^ metallized with carbon (Denton Vaccum, Moorestown, Nova Jersey, USA), and placed under a scanning electron microscope (JEOL JSM-6610LV, Akishima, Tokyo, Japan).

### Analysis by confocal laser scanning microscopy

After applying the hygiene protocols, the specimens were stained with a Live/Dead viability kit (SYTO-9 and propidium iodide) in the dark for 30 min, according to the manufacturers’ instructions, washed once, and resuspended in PBS for the reading under a Carl Zeiss LSM 800 Confocal Fluorescence Microscope with Airyscan.

### Statistical analysis

The normality of data was assessed by Shapiro-Wilk and the homoscedasticity was evaluated by Levene. Surface roughness, CFU/mL of AP, and XTT of the control group were analyzed by student’s t-test, whereas two-way ANOVA analyzed XTT and CFU/mL. Roughness before and after brushing and soaking cycles was assessed by one-way repeated measures ANOVA. Post hoc tests involved Tukey (homoscedastic data) and Games-Howell (heteroscedastic data). All analyses assumed a 5% significance level using SPSS software (version 20.0; SPSS Inc.). SEM and confocal microscopy were presented as descriptive analyses.

## Results

**Figure 2:**
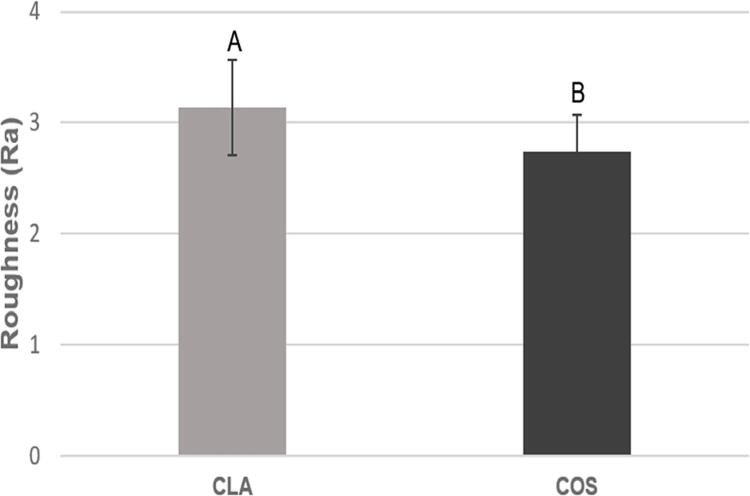
Mean surface roughness values (μm) of CLA and COS resin specimens used in the study and their respective standard deviations. Different capital letters denote significant difference (p=0.002) between the mean surface roughness of CLA and COS resins

**Figure 3:**
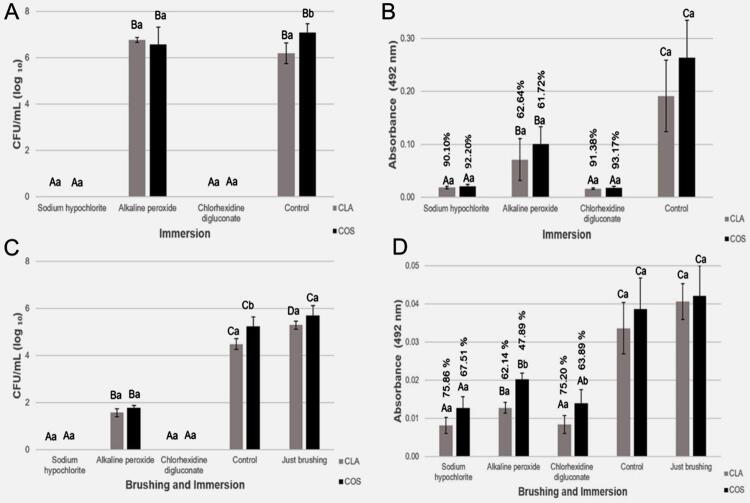
A, Mean log10 (CFU/mL) values obtained for immersion. C, Mean log10 (CFU/mL) values obtained for brushing and immersion. B, Mean optical density (OD) values obtained by immersion. D, Mean optical density (OD) values obtained by brushing and immersion. B, D, Percentage values on the top of the bars correspond to the percentage reduction in metabolism compared to the control group. Error bars: standard deviation (n=9). Different capital letters on the top of the bars denote significant difference (p<0.05) between immersion treatments for a same resin. Different lowercase letters denote significant difference (p<0.05) between resins for a same immersion treatment

Before the tests the mean surface roughness of CLA resin (Ra 3.13±0.42 μm) (Figure 2A) was higher (p=0.002) than that of COS (Ra 2.73±0.33 μm) (Figure 2B). CFU/mL of COS was greater than the one of CLA, regardless of the protocol adopted (immersion: p=0.036; brushing + immersion: p=0.00) (Figure 3A, C). Immersion in SH and CD, associated or not with brushing in a Lifebuoy solution, eliminated CFU/mL compared to the control group (p=0.00) (Figure 3A, C). AP immersion showed a CFU/mL similar to that of the control group (CLA: p=0.062; COS: p=0.615) (Figure 3A); however, when combined with brushing, it provided a lower CFU/mL (p=0.00) (Figure 3C). COS showed statistically higher CFU/mL than CLA compared to the control group during immersion and in brushing + immersion (p=0.007 and p=0.008) (Figure 3A, C).

The cellular metabolism (XTT) of COS specimens was statistically superior to that of CLA, regardless of the hygiene protocol adopted (immersion: p=0.006; brushing + immersion: p=0.00) (Figure 3B, D). Immersion in SH (CLA: p=0.001; COS: p=0.001) and CD (CLA: p=0.009; COS: p=0.001) (Figure 3B) and brushing + immersion in SH (CLA: p=0.00; COS: p=0.001) and CD (CLA: p=0.00; COS: p=0.003) (Figure 3D) reduced the XTT compared to the control group. AP alone as an immersion solution (CLA: p=0.009; COS: p=0.001) (Figure 3B) and associated with brushing (CLA: p=0.00. COS: p=0.003) (Figure 3D) a decrease in XTT was observed.

**Figure 4:**
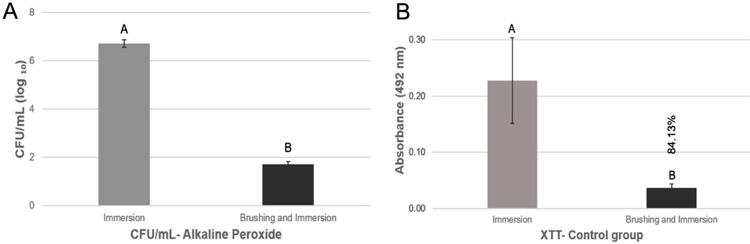
A, Mean log10 (CFU/mL) values obtained for immersion in alkaline peroxide and brushing and immersion in alkaline peroxide. B, Mean optical density (OD) values obtained by immersion and brushing and immersion in PBS. B, Percentage value on the top of the bar correspond to the percentage reduction in metabolism compared to immersion. Error bars: standard deviation (n=9). Different capital letters on the top of the bars denote significant difference (p<0.05) between treatments (p<0.05)

Regardless of the resin used, a comparison of the effectiveness of immersion in AP with brushing in a Lifebuoy solution + immersion in AP showed a CFU/mL reduction in the association (p=0.04) (Figure 4A). The XTT of the specimens subjected to immersion in PBS showed an 84.13% reduction (p=0.00) when exposed to brushing in Lifebuoy solution + immersion in PBS for both resins, compared to the specimens only subjected to immersion in PBS (Figure 4B).

**Figure 5:**
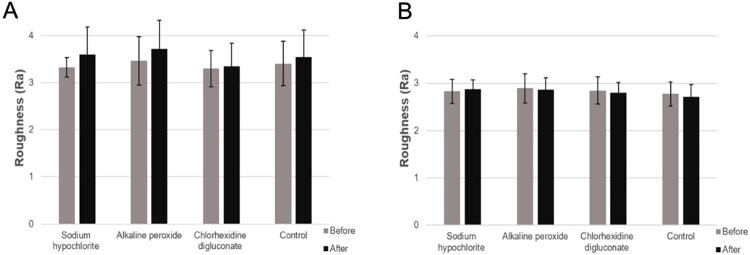
A, Mean roughness values (Ra µm) and respective standard deviations of CLA resin specimens before and after brushing and immersion in disinfectant solutions. B, Mean roughness values (Ra µm) and respective standard deviations of COS resin specimens before and after brushing and immersion in disinfectant solutions

Despite the solution employed, the roughness of CLA specimens statistically increased (p=0.008) after the combination of brushing and immersion (Figure 5A); however, it was reduced for COS after brushing and immersion in all evaluated solutions, except SH. The difference was not statistically significant (p=0.537) (Figure 5B).

**Figure 6:**
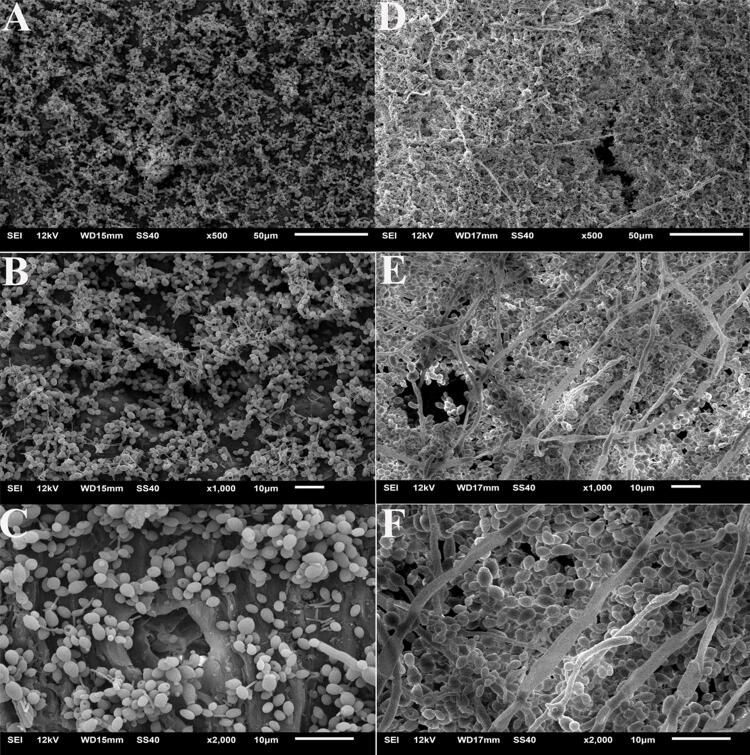
Scanning electron microscopic (SEM) images of the mature biofilm on the specimens A, CLA 500×. B, CLA 1.000×. C, CLA 2.000×. D, COS 500×. E, COS 1.000×. F, COS 2.000×

CLA showed a predominance of cells with yeast morphology and a slight presence of pseudohyphae and short hyphae (Figure 6A-C). Despite the predominance of yeasts, COS showed a significantly higher number of pseudohyphae and hyphae, including elongated hyphae (Figure 6D-F) and a more robust biofilm adhered to the specimen.

**Figure 7:**
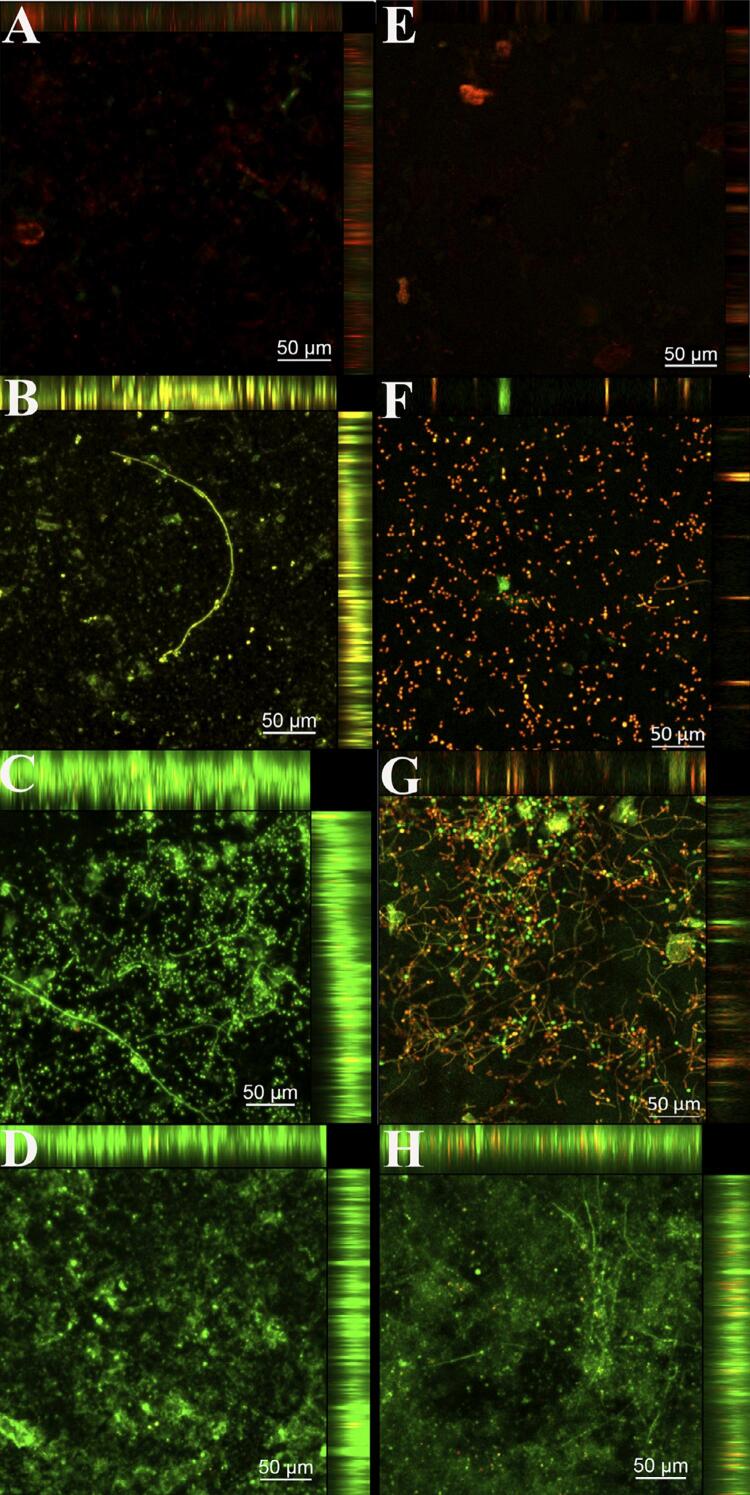
Confocal laser scanning microscopic images (excitation/emission of syto-9: 480/500 nm; propidium iodide: 490/635 nm) of mature biofilm on CLA and COS resin specimens after exposure to the brushing and immersion combination. A, CLA in sodium hypochlorite sodium. B, CLA in 2% chlorhexidine. C, CLA in alkaline peroxide. D, CLA in PBS-Control. E, COS in sodium hypochlorite sodium. F, COS in 2% chlorhexidine. G, COS in alkaline peroxide. H, COS in PBS-Control

Confocal laser microscopy was only performed for the groups of immersion associated with brushing, as the results of cell viability and metabolism presented that the AP was only effective with this association. The images obtained showed that brushing + immersion in SH (A-CLA, E-COS) (Figure 7) and CD (B-CLA, F-COS) (Figure 7) had a smaller biofilm thickness in both resins compared to other groups. Regarding SH, the reduction was almost total in the resins. A similar slight green fluorescence in 2% CD (Figure 7B, F) was still observed in both resins, whereas the elimination of dead cells (red fluorescence) (Figure 7F) was more difficult in COS. The specimens treated with brushing + immersion in AP (Figure 7C, G) showed a reduction in the biofilm compared to the control group. However, a relevant green fluorescence (viable cells) and hyphae were observed mainly for COS (Figure 7G). Although both resins showed similar biofilm thickness in the control group (D-CLA, H-COS) (Figure 7), COS (Figure 7H) revealed a more significant hyphae formation (Figure 7D).

## Discussion

We rejected the null hypothesis since the hygiene protocols influenced the biofilm formed on the specimens. Before the tests the mean roughness of the specimens, i.e., approximately 3.0 μm,^28^ was in accordance with the literature and simulated the internal surface of complete dentures.^29^ The COS roughness (Ra 2.73±0.33 μm) was lower than that of CLA (3.13±0.42 μm). Both resins showed mean roughness above 0.2 μm, which is the threshold for bacterial adhesion.^30^

Surface roughness influences the initial adherence of the microorganism since the irregular surface retains microorganisms and protects against shear forces, such as the mechanical cleaning of the denture.^28^ Despite the greater roughness of CLA, the biofilm formed on it showed cellular viability (CFU/mL) and metabolism (XTT) statistically lower than those of COS. We observed such behavior in provisional PMMA crowns, which showed a greater roughness but less accumulation of *Streptococcus mutans* than the printed resin.^31,32^ Meirowitz, et al.^33^ (2021) reported a greater adhesion of *C. albicans* after 4 hours of incubation on resin specimens for denture base made by 3D technology, compared to milled and conventional ones, even the printed specimens showed less roughness. Shim, et al.^22^ (2020) also observed that the less rough denture base specimens printed at 0° resulted in greater adhesion of *C. albicans* biofilm formed after 24 hours. These studies emphasize that although roughness is essential in initial adhesion of microorganisms on resin surfaces,^28^ other factors also influence, such as surface charge, surface free energy (SFE), hydrophobicity,^34^ structure, composition of the biomaterial,^35,36^and interactions among microorganisms.^28^ On the other hand, Osman, et al.^37^ (2023) found greater 24-hour biofilm formation of *C. albicans* on the rougher specimens 3D-printed at 45°, which differs from the specimens in our study, printed at 0°.

The building process of 3D-printed resins, layer-by-layer, results in grooves and pores on its surface that favors microbial adhesion.^22^ We did not evaluate surface hardness; however, it is an indicator of abrasion resistance. Thus, the greater hardness of the resin, the less susceptible it will be to surface scratches and damage when exposed to mechanical brushing and masticatory load, remaining smooth for longer and hindering *C. albicans* adhesion.^38,39^ Previous studies showed that 3D printed-denture base resins have lower hardness compared to PMMA-based resins.^40,41^ Alkaltham, et al.^41^ (2023) found that immersion in alkaline peroxide and sodium hypochlorite 1% for 180 days decreased the Vickers hardness of 3D-printed resins and did not change the hardness of PMMA. Thus, we hypothesize that the lower hardness of the 3D-printed resins, as well as the hardness reduction when these materials are immersed in disinfectant solutions, increases the susceptibility to the adhesion of microorganisms.

The biofilm formed on COS showed statistically higher viability and metabolism than CLA, despite the solution used and association or not with brushing. The presence of more CFU/mL in printed resins compared to heat-polymerized ones is supported by previous studies.^31-33^ By immersion alone or associated with brushing in a Lifebuoy solution, SH and CD eliminated CFU/mL and significantly reduced metabolism compared to the control group for both resins. The results from immersion in 0.25% SH on the reduction of CFU/mL of *Candida spp* have corroborated those from the literature.^11-13,26,42^ A 20-minute immersion in 0.25% SH reduced both the cellular metabolism of all evaluated species (*C. albicans, C. tropicalis and C. glabrata*) and the CFU/mL on PMMA^26^ specimens. Immersion of PMMA specimens contaminated with *C. albicans* biofilm in 1% SH for 10 seconds^15^ and 90 seconds^10^ reduced metabolism by 99.9%^15^ and 88%^10^, whereas that in 1% CD reduced the metabolism by 99.5%.^15^ Although the *C. albicans* biofilm formed on the COS showed higher viability and metabolism, the immersion solutions studied were effective in controlling the biofilm. However, we emphasize that recently published data showed that the immersion in SH 0.25%, CD 2%, and AP for the simulated period of 6 months significantly changed the color of the COS 3D-printed resin.^43^

This study showed that CD could not eliminate dead cells adhered to the specimens (Figure 6B, F), which is a relevant fact, since such cells remain adhered to the denture surface, acting as an adhesion agent for new microorganisms.^26^ CD reduces biofilm but SH does it more efficiently.^44^ The pH of the biofilm is close to neutral; therefore, higher or lower values alter both metabolism and surface properties of the microorganism, as well as the solid surface, increasing the electrostatic repulsion between the solid surface and the microorganisms and, consequently, the adherence of the microorganism to the surface.^45^ SH is an oxidizing agent with alkaline pH (>11) that influences the integrity of the cytoplasmic membrane, causing irreversible enzymatic inhibition, biosynthetic changes in cell metabolism, and degradation of phospholipids.^46^ CD is a cationic molecule, which binds to the cell of *C. albicans* causing severe degeneration to the cytoplasm, fragmentation, and desquamation of the cell wall, due to an osmotic imbalance of the cell, resulting in cell death.^9^ We hypothesized that, in this study, the neutrality of the CD pH possibly favored the permanence of cells adhered to the surface.^45^

The brushing efficiency was confirmed by an 84.13% metabolic reduction in specimens exposed to brushing + immersion in PBS compared to only immersion in PBS (Figure 4B). Although only immersion in AP did not reduce CFU/mL when compared to the control group, CFU/mL and XTT decreased when associated with brushing with Lifebuoy for both resins (Figure 4A). Immersion in AP reduced the XTT of microorganisms adhered to the specimens possibly due to the immediate damage of AP to the microorganisms, resulting in the metabolic reduction after the 3-hour incubation (XTT test). However, the damage was not irreversible and enabled the reproduction of the microorganisms after 48-h incubation. The literature corroborates this finding, since brushing associated with immersion in AP reduced CFU/mL, different from the groups subjected only to immersion in AP.^15^

In this study, after brushing and immersion cycles, regardless of the solution used, CLA showed an increase in surface roughness. Regarding printed COS, despite its tendency to reduce roughness, the difference was not statistically significant. Longer soaking and brushing cycles may result in more prominent differences in the behavior.

SEM revealed a more expressive presence of hyphal morphology and a more robust biofilm in COS compared with CLA. The polymorphism (yeasts, pseudohyphae, and hyphae)^47^ of *C. albicans* is an essential virulence factor; the hyphae presents a higher pathogenic potential since it applies mechanical force, helps penetration in the host’s epithelial tissue, damages endothelial tissues, and enables the spread of infection in the body’s bloodstream.^47^ The biofilm originated from hyphae of *C. albicans* is thicker and more resistant to removal than that from yeasts^47^. COS favored the presence of more hyphae compared to CLA, which explains the formation of a more robust biofilm, more difficult to be removed, with greater metabolism, more living cells after exposure to hygienic solutions, and more pathogenic.

The limitations of this study include the evaluation of only one 3D-printed denture base resin. The shape of the specimens differed from that of a denture base and the lack of hardness and color assessments could also be limitations. Future studies should evaluate both hydrophobicity and surface free energy of the resin to clarify the results obtained based on those factors.

## Conclusions

1. Before hygiene protocols, 3D-printed resin (COS) showed a thicker biofilm with more hyphae, despite its smoother surface. When exposed to hygiene techniques, it showed higher CFU/mL, XTT, biofilm thickness, and hyphae compared to the heat-polymerized resin (CLA), regardless of the immersion solution used.
2. Immersion in 0.25% SH and 2% CD, associated or not with brushing in Lifebuoy, were the most effective protocols regarding XTT and CFU/mL for both resins. SH also eliminated dead cells adhered to the specimens.
3. AP as immersion alone was not effective to reduce CFU/mL (48h), only when associated with brushing using Lifebuoy 0.78%.
4. The association of brushing with immersion was more effective than immersion alone in the control group (PBS) and AP.
